# Route and Regulation of Zinc, Cadmium, and Iron Transport in Rice Plants (*Oryza sativa* L.) during Vegetative Growth and Grain Filling: Metal Transporters, Metal Speciation, Grain Cd Reduction and Zn and Fe Biofortification

**DOI:** 10.3390/ijms160819111

**Published:** 2015-08-13

**Authors:** Tadakatsu Yoneyama, Satoru Ishikawa, Shu Fujimaki

**Affiliations:** 1 Department of Applied Biological Chemistry, The University of Tokyo, Tokyo 113-8657, Japan; 2 Soil Environment Division, National Institute for Agro-Environmental Sciences, Tsukuba, Ibaraki 305-8604, Japan; E-Mail: isatoru@affrc.go.jp; 3 Quantum, Beam Science Center, Japan Atomic Energy Agency, Takasaki, Gunma 370-1292, Japan; E-Mail: fujimaki.shu@jaea.go.jp

**Keywords:** cadmium, iron, metals in grains, metal speciation, metal transporter, rice (*Oryza sativa* L.), xylem-to-phloem transport, zinc

## Abstract

Zinc (Zn) and iron (Fe) are essential but are sometimes deficient in humans, while cadmium (Cd) is toxic if it accumulates in the liver and kidneys at high levels. All three are contained in the grains of rice, a staple cereal. Zn and Fe concentrations in rice grains harvested under different levels of soil/hydroponic metals are known to change only within a small range, while Cd concentrations show greater changes. To clarify the mechanisms underlying such different metal contents, we synthesized information on the routes of metal transport and accumulation in rice plants by examining metal speciation, metal transporters, and the xylem-to-phloem transport system. At grain-filling, Zn and Cd ascending in xylem sap are transferred to the phloem by the xylem-to-phloem transport system operating at stem nodes. Grain Fe is largely derived from the leaves by remobilization. Zn and Fe concentrations in phloem-sap and grains are regulated within a small range, while Cd concentrations vary depending on xylem supply. Transgenic techniques to increase concentrations of the metal chelators (nicotianamine, 2′-deoxymugineic acid) are useful in increasing grain Zn and Fe concentrations. The elimination of OsNRAMP5 Cd-uptake transporter and the enhancement of root cell vacuolar Cd sequestration reduce uptake and root-to-shoot transport, respectively, resulting in a reduction of grain Cd accumulation.

## 1. Introduction

Zinc (Zn) and iron (Fe) are required by plants in response to demands for plant growth, which is underlined by cellular metabolisms [1,2], while cadmium (Cd) is not essential for plants and is toxic at high levels. Likewise, Zn and Fe are essential in the human metabolism and their deficiency may lead to various diseases, while Cd is toxic and at high doses may even cause severe diseases like *Itai-Itai* disease (Table 1).

**Table 1:** Demands and toxicity of Zn, Cd, and Fe for plants and impacts of their dietary deficiency or excess on humans.

Metal	Demands/Toxicity for Plants	Deficiency Diseases/Excess Toxicity for Humans
Zn	Demand: Cofactor of over 300 enzymes including DNA- and RNA-polymerases; Excess toxicity: Unregulated binding of Zn to S-, N- and O-containing molecules	Deficiency: Stunting, diarrhea, pneumonia; Excess toxicity: Interference of Fe and Cu uptake in the intestines
Cd	Toxicity: Binding to protein SH-residues, exchanges with divalent cations such as Zn^2+^ and Ca^2+^, and excessive production of reactive oxygen species	Excess toxicity: *Itai-itai* disease (spinal and leg bone pain) caused by Cd accumulation in the liver and kidneys resulting in tubular renal dysfunction, osteoporosis, cancer, and cardiovascular diseases
Fe	Demand: Proteins involved in redox and electron transport. Leaf pigment formation; Toxicity: Free Fe can generate toxic levels of oxygen and hydroxyl free radicals through the Fenton reaction	Deficiency: Anemia, impaired mental development; Iron overload: Excessive accumulation of Fe in the liver, heart, and pancreas. Hemosiderosis; Hemochromatosis

Literature sources: Palmgren* et al.* [3], White and Broadley [4], and Clemens* et al.* [5].

Zinc and Fe in the grains of cereals such as rice are essential elements for humans. However, humans’ intake of Zn and Fe is generally not sufficient [3,6,7]. In contrast, Cd, which is sometimes contained at high levels in the rice grains grown in Cd-contaminated soils, is toxic for humans [8]. Therefore, it is necessary to enrich rice grains with Zn and Fe and to decrease their Cd content [9].

**Figure 1:**
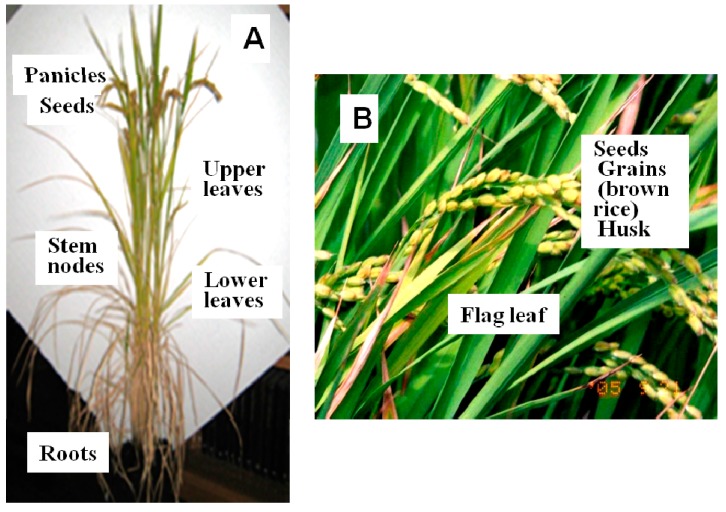
Rice plants (cv. Koshihikari) grown in potted soil (**A**) and in a field (**B**) at maturity.

Fukushima* et al.* [8] reported that, in the district where “*Itai-Itai* disease” was endemic, soil Cd levels ranged from 0.4 to 8 mg·kg^−1^ (dry soil) and those of brown rice were correlated, varying between 0.2 to 2 mg·kg^−1^ (dry weight), while in the case of Zn levels, although soil Zn levels varied greatly from 50 to 1200 mg·kg^−1^, those of brown rice had a much smaller range from 20 to 30 mg·kg^−1^. (Names of rice plant parts are shown in Figure 1).

Recent field studies in Thailand by Simmons* et al.* [10] on Cd, Zn, and Fe accumulation in rice grains (unpolished rice) under Cd/Zn contaminated soils, containing 2.9‒284 mg·kg^−1^ Cd, 254‒8036 mg·kg^−1^ Zn, and 0.5‒25 mg·kg^−1^ Fe, indicated that the harvested grains contained variable concentrations of Cd ranging from 0.02 to 5.0 mg·kg^−1^, but relatively constant concentrations of Zn (from 15 to 25 mg·kg^−1^) and Fe (from 7.5 to 12.7 mg·kg^−1^), although the concentrations of all three metals varied greatly in the stems and leaves. Thus, Zn and Fe levels in rice grains are little affected by the availability of Zn and Fe in the soil, while grain Cd levels vary greatly. Grain Zn concentrations in rice plants grown in the Philippines were unchanged by Zn fertilization (15 kg Zn·ha^−1^), while this Zn fertilization increased straw Zn concentrations by 43%‒95% [11].

When a rice cultivar (Todorokiwase) was grown in a culture solution at 0.01 to 3.0 mg·L^−1^ Cd, the Cd concentrations in the leaf blades greatly increased from 3 to 100 mg·kg^−1^ and those in the grains also increased from 0.5 to 10 mg·kg^−1^[12]. In contrast, when Zn concentrations in the culture solution were increased from 0.05 to 20 mg·L^−1^, Zn concentrations in the leaf blades greatly increased from 50 to 2500 mg·kg^−1^, but those in the grains increased only slightly from 33 to 80 mg·kg^−1^[13]. Recently, Jiang* et al.* [14] reported that when a rice cultivar (Handao297) grown in a nutrient solution containing Zn at 0.15 to 2250 µM, Zn concentrations in the shoots greatly changed from 53 to 1540 mg·kg^−1^, while those in brown rice changed only slightly from 52 to 106 mg·kg^−1^.

These results suggest that specific regulatory mechanisms for each of the three metals may exist between the xylem delivery to stems and leaves and the allocation of the metals to grains. In this review, to determine the regulation of the transport on Zn, Cd, and Fe via the xylem and phloem, we synthesized information of Zn, Cd, and Fe concentrations and their chemical forms in xylem and phloem saps, the localizations of metal transporters, and finally the routes of transport of these metals to the grains.

## 2. Uptake at Root-Surface Membranes and Radial Transport to the Xylem

Plant availability of metals from soils is controlled by three steps: (1) soil conditions (upland or flooded soil, soil solution pH); (2) mineralization (ionization and complex formation); and (3) uptake transporters.

**Figure 2:**
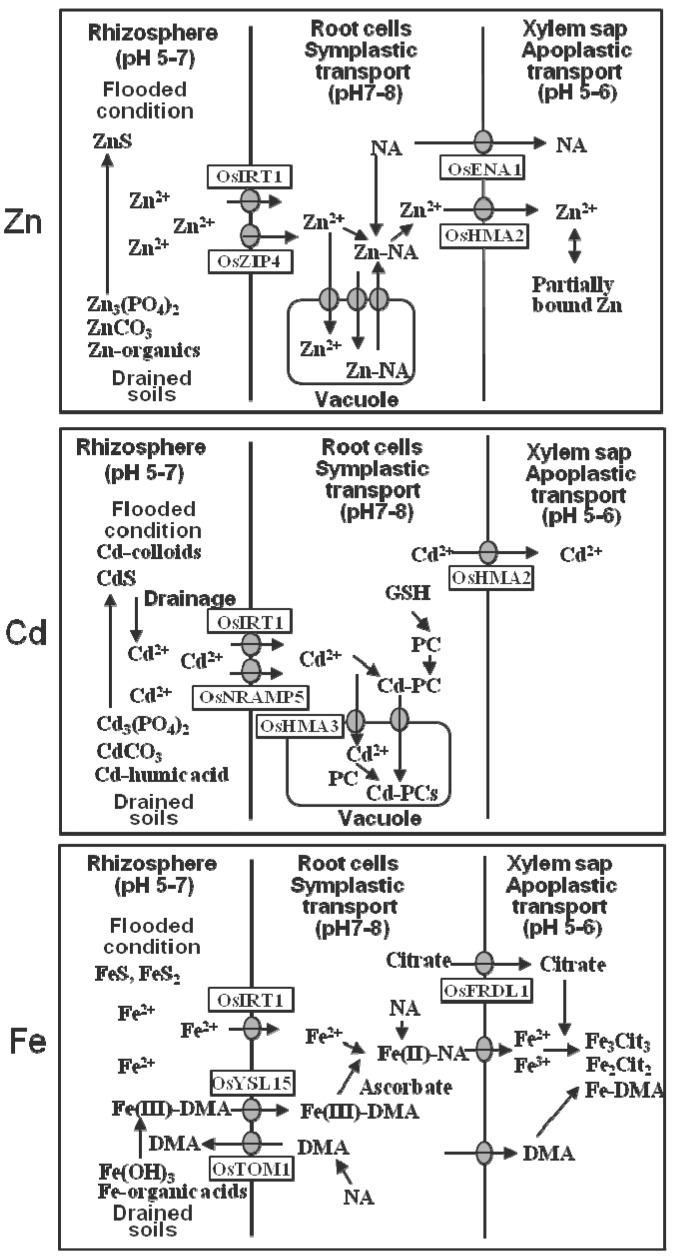
Models of uptake and transport of Zn, Cd, and Fe in rice roots. DMA, 2′-deoxymugineic acid; GSH, glutathione; NA, nicotianamine; PC, phytochelatin. Arrows indicate flows of substances and grey ellipses indicates transporters.

As shown in Figure 2, Zn in both drained and flooded soils is largely ions (Zn^2+^), although some Zn may be bound to organic substances, and immobilized as Zn–sulfide (ZnS) in the anaerobic layer [15]. Cadmium in drained acidic soils is ionized as Cd^2+^, while Cd in paddy alkaline soils is present in the forms of CdCO_3_ and humic acid-bound Cd [16]. Cd is immobilized as Cd-sulfide (CdS) and colloids-bound Cd by flooding of soils [17]. Drainage converts CdS to Cd^2+^ and dramatically increases its availability to plants. Fe in acidic soils is ionized as Fe^2+^/Fe^3+^, while in aerobic alkaline soils Fe is immobilized as Fe(OH)_3_.

**Table 2:** Transporters involved in the acquisition and inter-organ transport of Zn, Cd, and Fe in rice plants.

Site	Zn	Cd	Fe
Acquisition at root cell membranes	OsIRT1 ^1^; OsZIP4,5 ^2^	OsNRAMP5 ^3^; OsIRT1/OsIRT2 ^4^	OsYSL15 ^5^; OsIRT1/OsIRT2 ^6^
Vacuolar import through the tonoplast in root cells	(ZIF1) ^7^	OsHMA3 ^8^	
Xylem loading at root pericycle cells	OsHMA2 ^9^	OsHMA2 ^5,9^	
Xylem-to-phloem transport at nodes	OsHMA2 ^10^	OsHMA2 ^10^	OsYSL16 ^11^
Phloem loading after mobilization in leaves	ZIP and YSL families		OYSL15 ^12^
Phloem unloading at reproductive organs			OsYSL18 ^13^

^1^ Lee and An [18]; ^2^ White and Broadley [4]; ^3^ Ishikawa* et al.* [19], Sasaki* et al.* [20]; ^4^ Nakanishi* et al.* [21]; ^5^ Inoue* et al.* [22]; ^6^ Ishimaru* et al.* [23]; ^7^ Haydon* et al.* [24]; ^8^ Ueno* et al.* [25]; ^9^ Nocito* et al.* [26], Takahashi* et al.* [27]; ^10^ Yamaji* et al.* [28]; ^11^ Kakei* et al.* [29]; ^12^ Lee* et al.* [30]; ^13^ Aoyama* et al.* [31].

As shown in Table 2, the uptake of these metals at root-surface membranes is driven by specific uptake transporters, Zn^2+^, Cd^2+^, and Fe^2+^ commonly by OsIRT1 (rice iron-regulated transporter1) [18,21,23] and Cd^2+^ predominantly by OsNRAMP5 (rice natural resistance-associated macrophage protein5) [19]. The possible uptake of some Zn–phytosiderophores together with the uptake of Zn^2+^ by a Zn-deficiency-tolerant line of rice has been suggested under lowland conditions [32]. Iron (Fe^3+^) in neutral and alkaline soils binds to mineral crystals and organic substances so strongly that such soil ions are barely available to plants and the iron is solubilized by forming a complex with a phytosiderophore, 2′-deoxymugineic acid (DMA) [33], which is excreted by rice roots by OsTOM1 (rice DMA effluxer) [34]. Fe(III)–DMA complexes in the soil solution are taken up by OsYSL15 (rice yellow stripe-like15) [22]. Under flooded conditions, rice plants may absorb both Fe(III)–DMA and Fe^2+^ [23].

Zinc (Zn^2+^) and Fe^2+^ in root cell cytosols form complexes with nicotianamine (NA) in the forms of Zn–NA and Fe(II)–NA (Figure 2). Cadmium in the root cell cytosols may be Cd^2+^ ions or complexes with phytochelatins (PCs). Fe(III)–DMA absorbed in the root cell cytosols can be reduced by ascorbate, transforming to Fe(II)-NA [35]. Fe of cytosolic Fe(II)–NA may be excreted to the xylem and makes complexes predominantly with citrate (Fe_2_Cit_2_, Fe_3_Cit_3_) and some with DMA (Fe–DMA) [36].

Root cell vacuoles can function as a storage compartment for metals. Zn–NA in the root cell cytosols is transferred to the vacuoles by the ZIF1 (zinc induced facilitor1) transporter [24]. Cadmium in the cell cytosols may be partly sequestered through the tonoplast as Cd^2+^ by OsHMA3 (rice heavy metal ATPase3) [25] and as Cd-PC complex, presumably by ABCC-type transporters in *Arabidopsis* [37], and both are stored as Cd-PCs in the vacuoles. Enhancement of OsHMA3 activity has been found to increase storage in roots and decrease the transport of Cd to the shoot and the final accumulation of Cd in rice grains [25]. A high rate of root-to-shoot transport and subsequent accumulation in the grains of ^107^Cd, which was administered from a culture solution, was observed in OsHMA3-depleted rice lines [38].

OsHMA2, which localizes at the root pericycle, is a major transporter of Zn and Cd for xylem loading [26,27]. The excretion of citrate from the root cells to the xylem is partly operated by OsFRDL1 (rice ferric reductase defective1-like) to enhance Fe-transport in the xylem as Fe(III)–citrate complex [39].

## 3. Xylem and Phloem Transport

The concentrations of Zn, Cd, and Fe and their chemical forms in rice xylem and phloem saps from 28- to 33-day-old vegetative plants were analyzed. The phloem sap was collected by a unique and excellent technique using the insect-laser method as described by Kawabe* et al.* [40]. Briefly, the insects used were *Nilaparvata lugens* Stål (brown planthopper). Rice plants at the 8th to 9th leaf-age were transferred to 500 mL plastic bottles, one plant per bottle, and placed in an artificially lit chamber (130 µmol·m^−2^·s^−1^ photosynthetically active irradiation, 12 h light and 12 h dark cycle). After the plants’ adaptation to the environment over 1 or 2 days, overhead projector film sheet cylinders (3 cm high, 1 cm in diameter) with cotton stoppers on both sides were set on the sheaths of the fully developed leaves, and female insects, that had been forced to fast overnight, were transferred into the cylinders to allow the insects to suck the phloem sap. After 1–3 h, the insects’ stylets were cut by a YAG laser (SL129Nd; NEC, Tokyo, Japan) beam. After the phloem sap was collected, shoots were cut with a knife at 2 cm from the root-shoot interface and the exudates (xylem sap) from the cut surfaces were collected.

**Figure 3:**
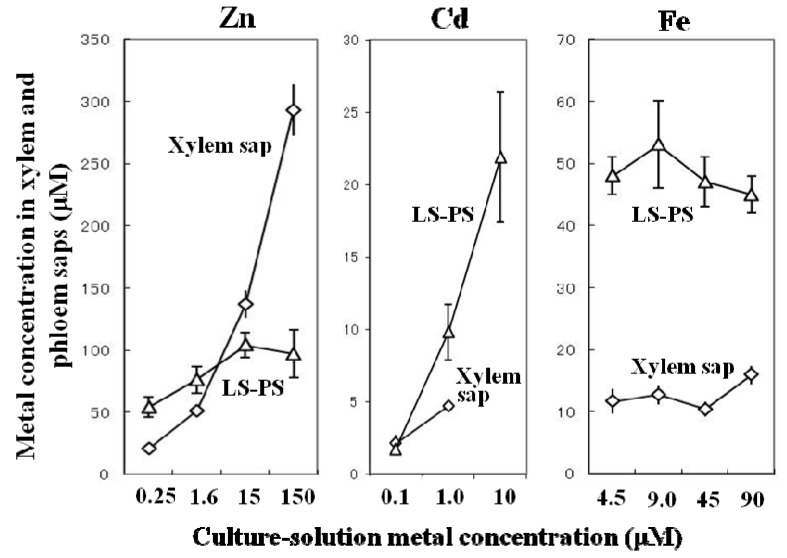
Metal concentrations in the xylem and phloem saps of vegetative rice plants under various culture-solution metal concentrations. Phloem sap was collected from the leaf sheaths (LS-PS) of the 7th leaves. Data for Zn and Fe are from Nishiyama [41], and data for Cd are from Mariyo Kato [42].

The Zn concentrations in the xylem and phloem saps under four different Zn concentrations in the culture solution are shown in Figure 3. The Zn concentrations in the xylem sap increased with those in the culture solution, while the Zn concentrations in the phloem sap varied only slightly between 55 and 100 μM. With 0.1 µM culture-solution Cd, the xylem and phloem Cd concentrations were around 20-fold higher than the culture-solution Cd concentrations. Under treatment with 10 µM Cd, xylem sap was not exuded due to wilting. Cd concentrations in the collected phloem sap increased with the medium Cd concentrations (Figure 3). Although the Fe concentrations in the culture solution ranged greatly at 4.5, 9.0, 45 and 90 µM, xylem Fe concentrations remained nearly constant at 12 µM under the lower three culture-solution Fe treatments and 16 µM under the 90 µM treatment (Figure 3). Phloem-sap Fe concentrations, around 50 µM Fe, were not affected by the different Fe treatments.

The chemical forms in xylem and phloem saps from young rice plants are shown in Table 3. Using the phloem and xylem saps collected as described above, chemical forms (speciation) of Zn, Cd, and Fe in such saps were investigated using size-exclusion high performance liquid chromatography (SE-HPLC, Hitachi, Tokyo, Japan), inductively coupled plasma-mass spectrometry (ICP-MS, Yokogawa, Tokyo, Japan), capillary electrophoresis coupled to mass spectrometry (CE-MS, Agilent Technologies, Walbronn, Germany), and electrospray ionization time-of-flight mass spectrometry (ESI-TOF MS, JOC, Tokyo, Japan).

The Zn chemical forms in xylem sap are free ions and Zn partially bound to unidentified chelators [39], while the Zn in phloem sap was dominantly bound to NA [43]. The Cd chemical form in the xylem sap is primarily in free ions (Mariyo Kato [42]), while the Cd in the phloem sap was largely bound to specific proteins although some Cd was bound to low-molecular thiol compounds [44]. The Fe chemical forms in xylem sap were found to be bound largely to citrate (around 65%) and slightly to DMA (around 5%) [36], while the Fe in phloem sap bound to DMA, citric acid, and proteins [43]. Recently, metal species in the xylem and phloem saps from various plants including rice were summarized by Álvarez-Fernández* et al.* [45].

**Table 3:** Chemical forms of Zn, Cd. Fe, and Cu in xylem and phloem saps from rice plants. Metal speciation analyses in xylem and phloem saps were conducted by using SE-HPLC, ICP-MS, CE-MS, and ESI-TOF MS.

Metal	Chemical Forms in Xylem Sap (pH 6)	Chemical Forms in Phloem Sap (pH 8)
Zn	Free ions and partially bound ^1^	Bound dominantly to NA ^2^
Cd	Primarily in free ions ^3^	Bound largely to specific proteins and slightly to thiol-compounds ^4^
Fe	Bound largely to citrate (around 65%) and slightly to DMA (around 5%)and some in free ions ^5^	Bound to DMA, citrate, and proteins ^2^
Cu	Bound dominantly to DMA ^6^	Bound to NA, histidine and proteins ^6^

^1^ Nishiyama [41]; ^2^ Nishiyama* et al.* [43]; ^3^ Mariyo Kato [42]; ^4^ Kato* et al.* [44]; ^5^ Ariga* et al.* [36]. ^6^ Ando* et al.* [45].

Changes in copper (Cu) concentrations in xylem and phloem saps and the chemical forms of Cu in rice xylem and phloem saps were investigated by Ando* et al.* [46]. Xylem Cu concentrations were found to range slightly between 5 and 8 µM when culture-solution Cu concentrations ranged from 0.16 to 1.28 µM and the Cu in xylem sap was dominantly bound to DMA. Phloem sap Cu concentrations ranged between 21 and 43 µM and the Cu in phloem sap was bound to NA, histidine and proteins.

Based on the results on variation in metal concentrations (Figure 3) and their chemical forms in xylem and phloem saps (Table 3), the following interesting relationship regarding the regulatory mechanisms that underlie the different changes in the Zn, Cd, Fe, and Cu concentrations in both saps is suggested; if xylem sap contains substantial free metal ions as in the cases of Zn and Cd, its metal concentration increases with increasing culture-solution metal concentration, while if xylem sap contains metal–ligand complexes as in the cases of Fe and Cu, its metal concentration remains nearly constant. If the metals in the phloem saps are definitely bound to ligands as in the cases of Zn–NA, Fe–DMA, and Cu–NA, the metal concentration remains quite constant. The concentration of NA, which may bind Zn and Cu in rice phloem sap, was around 70 µM [43,45], and that of DMA, which may bind Fe in rice phloem sap, was around 150 µM [43]. However, phloem Cd is apparently an exception; phloem concentrations changed with culture-solution and xylem-sap Cd concentrations (Figure 3). The major form of Cd in the phloem saps is protein-bound and the Cd-binding capacity of the proteins may be sufficient to load all Cd in the phloem sap [44].

**Figure 4:**
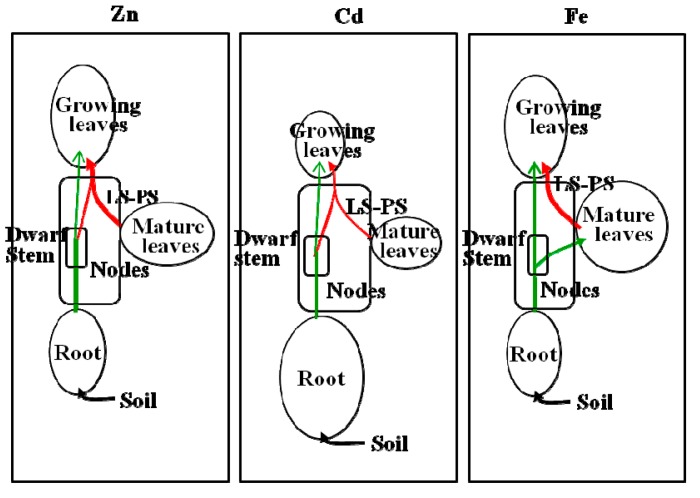
Models of the transport of Zn, Cd, and Fe via xylem and phloem in rice plants at the vegetative stage. “Leaves” are leaf blades. “Stem” includes the dwarf culm and leaf sheaths. The green line shows xylem transport. The red line shows phloem transport. Phloem sap was collected from the leaf sheaths (LS-PS). Information is from Yoneyama* et al.* [55]. Important note: At the vegetative nodes of the dwarf stem, Zn and Cd ascending xylem (green line) are transferred to the phloem (red line) via the xylem-to-phloem transfer system at the nodes, while Fe in the xylem (in the form of Fe–citrate) does not pass through this system.

**Figure 5:**
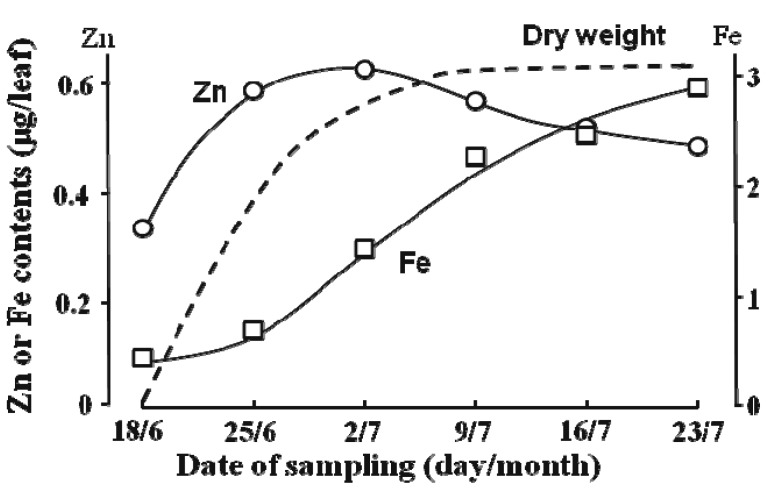
Changes in Zn and Fe amounts in the blade of the 10th leaf along with dry weight growth. (Adapted from Obata and Kitagishi [49]). The dry weight of the leaf was drawn arbitrarily as the dry weight at 23/7 had reached the maximum. **○**: change in the content of Zn; **□**: change in the content of Fe. (Adapted from Obata and Kitagishi [49]).

With respect to the transport of Zn in xylem sap to rapidly growing leaves, Obata and Kitagishi [47] indicate that some Zn ascending via the xylem (transpiration stream) is transferred to the phloem at the vegetative nodes in addition to the Zn which is mobilized from mature leaves (Figure 4). Together this Zn with different origins is terminated to the intercalary meristem to synthesize ribosomes [48]. Such active transport through both routes supports the high Zn accumulation at the meristem, which has been reported to reach 300 mg Zn·kg^−1^ dry weight (DW) in the rapidly growing leaf part as compared to 30 mg Zn·kg^−1^ DW in the mature leaves [49]. Figure 5 shows this unique and quick accumulation of Zn in the early stage of leaves compared to the slow accumulation of Fe and also the early initiation of the decrease after the peak of Zn content, which was one week before reaching the DW peak [49]. The amount of Zn transferred to the phloem via the xylem-to-phloem transfer system seems to be regulated and constant: the transfer rate of Zn from xylem sap is relatively high when the xylem sap Zn concentration is low, and low when the xylem sap Zn concentration is high [50]. The ratio of the Zn deprived by the xylem-to-phloem transfer to that remobilized from the leaves is estimated to be around 3:7 [49]. Partitioning of Cd to the top may be similar to that of Zn and the vegetative nodes may also be the site of the xylem-to-phloem transfer of Cd [51]. ^115m^Cd absorbed by roots is partitioned to the dwarf stem and leaves by the pattern of accumulation in the nodal part of the stem, and transport is then predominant to the rapidly growing leaves with little to the mature leaves [52], similar to that of ^65^Zn [50]. Recently, preferential movement of ^109^Cd and ^45^Ca, which were administered to a root–bathing solution as ^109^CdCl_2_ and ^45^CaCl_2_, to the newest leaf by transfer to the phloem at the stem, was confirmed by radioisotope imaging [53]. The rate of Fe involved in xylem-to-phloem transfer could be smaller than that of Zn, since the demand-time of Fe is late compared to that of Zn (Figure 5, [49]). It is interesting to note that Fe in rice xylem sap has been found to contain small amounts of Fe–DMA and large amounts of Fe–citrate while the Fe in the xylem sap of barley plants includes a considerable fraction of Fe–mugineic acid (MA) [36]. Barley plants can transfer such xylem sap Fe–MA to the young leaves through the xylem-to-phloem system at nodes as revealed by ^52^Fe tracing [54].

Xylem transfer cells found in vegetative nodes [56,57,58] and localized metal transporters (Table 2) may support the active xylem-to-phloem transfer of xylem sap Zn and Cd.

## 4. Accumulation of Zn, Cd, and Fe in Rice Grains

**Figure 6:**
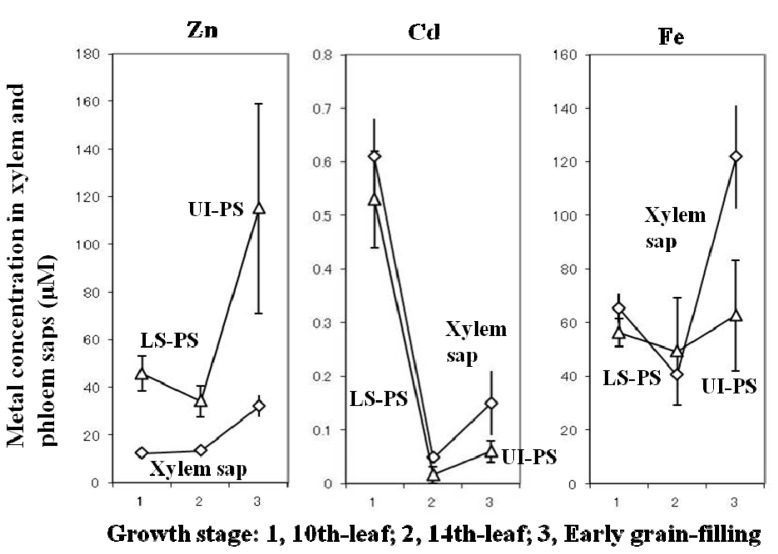
Metal concentrations in the xylem and phloem saps obtained from rice plants grown in a flooded Cd-contaminated soil. Phloem sap was collected from the leaf sheaths (LS-PS) of the 9th leaves at 10th-leaf stage and the 13th leaves at 14th-leaf stage, and from the uppermost internodes (UI-PS) at early grain-filling. Data are from Yoneyama* et al.* [55]. ◊: metal concentrations in the xylem sap, Δ: metal concentrations in the phloem sap.

**Figure 7:**
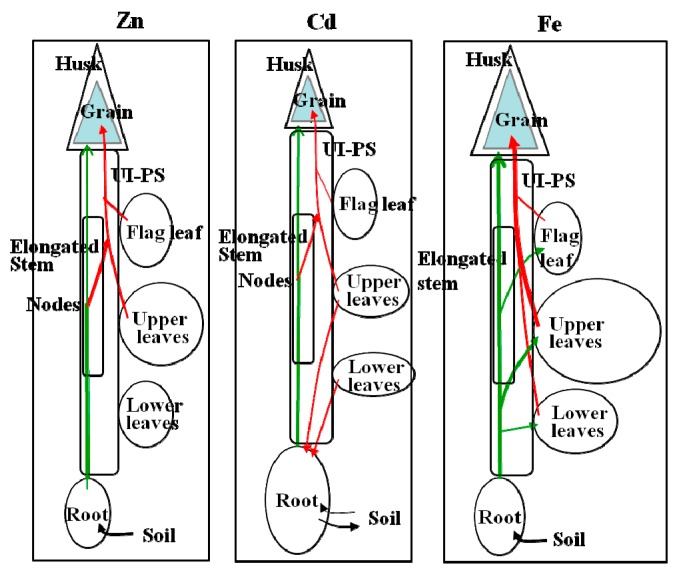
Models of the transport of Zn, Cd, and Fe via xylem and phloem in rice plants at grain filling. “Leaves” are Leaf blades. “Stem” includes elongated culm, leaf sheaths, and panicles except husk and grain. Green line shows xylem transport. Red line shows phloem transport. Phloem sap was collected from the uppermost internode (UI-PS). Information is from Yoneyama* et al.* [55]. Important note: At the nodes in the elongated stem, Zn and Cd ascending xylem (green line) are transferred to phloem (red line) via the xylem-to-phloem transfer system, while Fe in the xylem (in the form of Fe–citrate) does not pass through this system.

**Figure 8:**
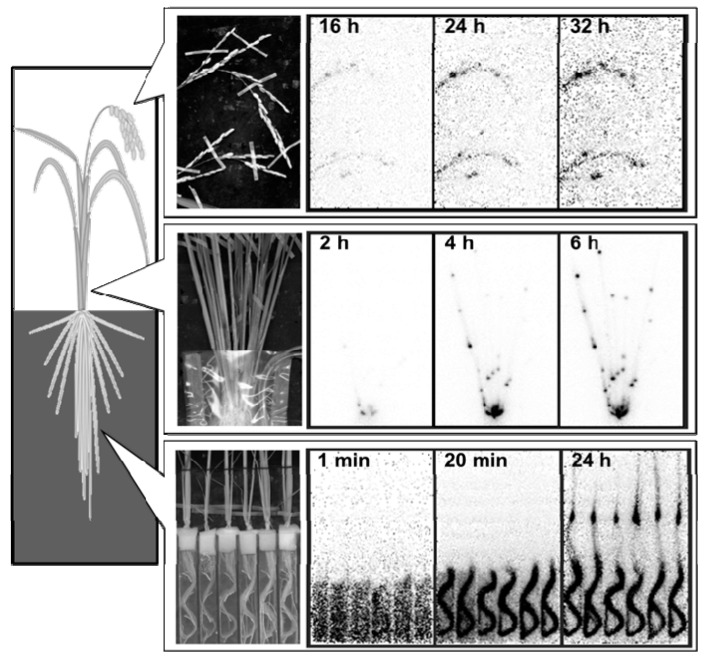
Movement of ^107^Cd in rice plants administered from the root–bathing solution visualized with a positron-emitting tracer imaging system (PETIS). Regenerated from the original data of Ishikawa* et al.* [38] and Fujimaki* et al.* [62]. Important note: The images show the uptake and accumulation of ^107^Cd by roots, transport to the shoots accompanying accumulation at the nodes (probably occurring during xylem-to-phloem transport), and final destination in the grains.

Phloem and xylem saps were collected from rice plants grown in Cd-contaminated flooded soil. Phloem sap was obtained from leaf sheaths at the 10th and 14th leaf ages and from the uppermost internodes at the grain-filling stage (Figure 6 [55]). Phloem loading after both remobilization from the leaves and stems and xylem-to-phloem transfer at stem and panicle nodes of the currently ascending fluids may be the source of grain Zn [55,59,60] and Cd [55,61,62], but for Fe the route of remobilization from other organs may be dominant as shown in Figure 7. The contribution of the xylem-to-phloem transfer rate of Zn and probably also of Cd is evident from the high and increased Zn concentration in phloem sap collected at the uppermost internode as compared to that collected from leaf sheaths at the 14th-leaf stage; in contrast, no such increase was observed for Fe (Figure 6). A positron-emitting ^107^Cd imaging study of hydroponically grown rice plants at the panicle-initiation stage suggests the highly preferential transport of currently absorbed Cd to the panicles through the presumed xylem-to-phloem transfer at the nodes (Figure 8) [63]. The presence of xylem transfer cells in the nodes [56] and in the spikelet nodes at the region of the rachilla and pedicle [64,65] and metal transporters, OsHMA2 for Zn and Cd [28] and OsYSL16 (rice yellow stripe-like16) for Fe–DMA [29] although the fraction of Fe–DNA is small [36], may be involved in the active xylem-to-phloem transfer of minerals (at the nodes) currently ascending via the xylem from the roots. The amount of transfer of Zn via the xylem-to-phloem system may be highly regulated at a certain level where there is a large supply of Zn through the xylem, as shown in wheat detached panicles [66]. In contrast, such a restriction may not work in the transport of Cd, since the Cd concentration ascending the xylem is much smaller than that of Zn. Jiang* et al.* [67] demonstrated the efficient transport to the grains and little partitioning to the leaves of ^65^Zn applied to rice plants from a culture solution during the flowering period. Rodda* et al.* [62] estimated that 60% of the final grain Cd could be attributed to remobilization from other tissues and the rest (40%) from uptake during grain maturation. Importantly, mineral transporters for xylem-to-phloem transport at the nodes (OsHMA2, OsYSL16), for the phloem loading at leaves (OsYSL15), and for unloading at panicles (OsYSL18) may play important functions in the regulation of metal transport (Table 2).

The deposition of metals in grains is revealed as metal partitioning: Zn and Fe, which combine with phytic acid, are largely accumulated in the aleurone cell layer [4,68] but their concentrations are reduced in white rice by polishing, while a large proportion of Cd is localized at the storage protein (glutelin) in the endosperm [69] and the Cd concentration in white rice is unchanged by polishing.

As stated in the Introduction section above, the Zn concentration in rice grains is affected only slightly by the availability of culture-solution metals, as compared to the great changes in Zn concentrations in the leaves and stems [13,14]; grain Cd concentrations are easily affected [12]. This observation is strongly related to the regulated and small changes in phloem concentrations of Zn, which may be in the form of Zn–NA complexes. In contrast, Cd loaded into phloem fluids may quickly bind to specific Cd-binding proteins, and those are unsaturated with Cd and sufficiently present relative to Cd, as described by Kato* et al.* [44]. Therefore, there are fewer limitations in the phloem transport of Cd than in Zn phloem transport.

The Fe in phloem sap may be derived largely from remobilization at the leaves (Figure 7, [55]) and probably bind to DMA and some citrate and proteins [43]. Therefore, the amount of Fe transport to the grains may be regulated by ligands and chelators. Schuler* et al.* [70] reported that in *Arabidopsis thaliana*, Fe–citrate in xylem sap may be delivered to the mature leaves and then Fe may be carried to the reproductive organs via the phloem as Fe–NA from the mature leaves. In the case of rice plants, Fe in the xylem is largely bound to citrate with slight binding to DMA [36]. Therefore, a large fraction of xylem Fe may be delivered first to the leaves as Fe–citrate and then to the seeds via the phloem, probably as Fe–DMA. It is possible that small amounts of Fe–DMA in rice xylem sap [36] travel through the xylem-to-phloem transport system at nodes by the OsYSL16 transporter [29]. Foliar application of a solution containing NA in addition to Fe and amino acids to a field-grown rice plant was found to increase the Fe and Zn concentrations in brown rice over those under a treatment with a solution containing Fe and amino acids [71].

## 5. Molecular Technologies to Increase Grain Fe and Zn and to Reduce Grain Cd

Recent attempts at the biofortification of Fe and Zn in rice grains using transgenic techniques have produced interesting results. The introduction of a yeast ferric chelate reductase gene to rice plants induced the enhancement of Fe uptake and an increase in grain yields but had no effect on Fe and Zn concentrations in the grains [72]. In contrast, the over-expression of the barley NA synthase gene *HvNAS1* in rice plants caused an increase in DMA and NA concentrations in roots, shoots, and seeds as well as of Fe, Zn, and Cu concentrations in grains [73]. These results are consistent with the proposed principle that the phloem-sap and grain concentrations of metals may be regulated by making use of the nature of metal-chelating compounds. It is valuable to note that high-NA grain may increase iron-bioavailability to mammals and humans [74,75].

There are three possible ways to reduce Cd content in rice grains: (1) the selection of lines/varieties with low Cd in phloem sap [44]; (2) more sequestration of Cd in the root–cell vacuoles by overexpression of OsHMA3 [25]; and (3) elimination of a Cd-uptake transporter (OsNRAMP5) in the root membranes [19]. Ishikawa* et al.* [19] report that carbon ion beam irradiation produced Cd-deficient rice mutants (Koshihikari-Kan1) with <0.05 mg Cd·kg^−1^ in the grains compared with a mean of 1.1 mg Cd·kg^−1^ in the parent, cultivar Koshihikari (a high eating-quality rice) when plants were grown in Cd-contaminated fields, whereas their Zn and Fe concentrations were less affected (Table 4), and they also developed DNA markers to select cultivars carrying *osnramp5*. In terms of human intake of Cd from Cd-contaminated foods such as rice, its bioavailability and retention in the liver and kidneys are dramatically increased due to the deficiency of Zn, Fe, and Ca in foods [76]. Therefore, biofortification of Zn, Fe and Ca in the grains is highly essential to reduce human Cd intake.

**Table 4:** Concentrations of Zn, Cd and Fe of grains of cultivar Koshihikari (Koshi) and its OsNRAMP5-depleted mutant Koshihikari-Kan1 (Kan1) grown on three Cd-contaminated soils whose irrigation was treated with prolonged drainage after the early-period flooding. Data are means of 5 samples. ND, not detected. Data are from Ishikawa* et al.* [19].

Field (Soil Cd mg·kg^−1^)	Grain Zn (mg·kg^−1^)		Grain Cd (mg·kg^−1^)		Grain Fe (mg·kg^−1^)	
	Koshi	Kan1	Koshi	Kan1	Koshi	Kan1
A (1.35)	48.8	36.9	0.57	ND	21.5	16.4
B (1.21)	34.4	31.4	1.86	0.02	14.2	15.8
C (0.35)	50.4	37.5	0.97	0.03	15.1	14.3

## 6. Conclusions

Zn and Fe are essential nutrients for both plants and humans but Cd is non-essential and toxic if it accumulates at high levels for plants and, more severely, for humans. All three metals are contained in the grains of rice, a staple cereal worldwide. Several studies have reported that Zn and Fe concentrations in rice grains harvested under different levels of soil or hydroponic metals are known to change only within a small range, while grain Cd concentrations show greater changes and easily reach the allowable maximum in polished rice (0.4 mg·kg^−1^) [77]. To gain insight into the mechanisms underlying such different metal contents, we synthesized information about the routes of metal transport and accumulation in rice plants, finding novel regulations involving metal speciation in xylem and phloem saps, metal transporters, and the xylem-to-phloem transport system. Specific metal transporters are involved in uptake in the roots, delivery to the shoots via the xylem-to-phloem transport system, and final transport to the grains via the phloem. Xylem sap Zn is in free ions and partially bound to unidentified chelators and has a highly variable concentration, while phloem sap Zn is dominantly Zn–NA complex with a highly regulated concentration. Xylem sap Cd is primarily in free ions with a variable concentration, while phloem sap Cd is bound to proteins and thiol compounds, and its concentration is dependent on xylem sap Cd. Cd loaded into phloem fluids may quickly bind to specific Cd-binding proteins, those are unsaturated with Cd and sufficiently present relative to Cd. Xylem sap Fe (largely as Fe–citrate) is allocated to all leaves, while phloem Fe is bound to DMA, citrate, and proteins. At grain-filling, Zn and Cd ascending in xylem sap are transferred to the phloem by the xylem-to-phloem transport system operating at stem and panicle nodes, where xylem transfer cells and specific metal transporters are recognized. Grain Fe is largely derived from the leaves by remobilization. Thus, the concentrations of grain Zn and Fe are highly regulated within a small range, while that of grain Cd varies depending on xylem supply. Transgenic techniques to increase tissue and phloem-sap concentrations of NA and DMA are useful to increase grain Zn and Fe concentrations. The elimination of OsNRAMP5 Cd-uptake transporter and the enhancement of root cell vacuolar Cd sequestration reduce uptake and root–to-shoot transport, respectively, resulting in a great reduction of Cd accumulation in grains.
